# Correlation between the efficacy of stem cell therapy for osteonecrosis of the femoral head and cell viability

**DOI:** 10.1186/s12891-020-3064-4

**Published:** 2020-01-29

**Authors:** Zhan Yu Wu, Qi Sun, Ming Liu, Brian E. Grottkau, Zhi Xu He, Qiang Zou, Chuan Ye

**Affiliations:** 1 Department of Orthopaedics, The Affiliated Hospital of Guizhou Medical University, Guiyang, China; 2 Center for Tissue Engineering and Stem Cells, Guizhou Medical University, Guiyang, China; 3 Yueyang Traditional Chinese Medicine Hospital, Hunan, China; 4 Department of Orthopaedics, West China Hospital of Sichuan University, Chengdu, China; 5 Department of Orthopedics, Massachusetts General Hospital, Boston, MA USA; 6 Key Laboratory of Adult Stem Cell Transformation Research, Chinese Academy of Medical Sciences, Guiyang, 550004 China; 7 China Orthopaedic Regenerative Medicine Group (CORMed), Hangzhou, China

**Keywords:** Osteonecrosis of the femoral head, Bone marrow mesenchymal stem cells, Curative effect

## Abstract

**Background:**

Osteonecrosis of the femoral head (ONFH) is a common disease that greatly affects the quality of life of patients. Repair of the necrotic area is key to successful treatment. Currently, the combination of stem cell transplantation and decompression is used clinically to promote the repair of necrotic areas based on the characteristics of stem cells. However, a considerable number of patients do not achieve a satisfactory outcome in terms of repair of the femoral head necrotic area, and it is very important to determine the reasons for the poor curative effect. The aim of this study was to investigate the correlation between stem cell viability and the repair efficacy of stem cell therapy combined with core decompression for early-stage ONFH.

**Methods:**

A total of 30 patients with idiopathic ONFH underwent core decompression combined with autologous stem cell transplantation. The Harris hip score (HHS) and difference in necrosis area before and after surgery were measured. The mean repair ratio was set as the threshold to divide the patients into group A (ratio above the mean) and group B (ratio below the mean). The ultrastructure, proliferative capacity, and multidirectional differentiation ability were compared between the groups.

**Results:**

At 9 months after surgery, the HHS and magnetic resonance imaging (MRI) findings improved by varying degrees. Based on the mean repair ratio of (62.2 ± 27.0)%, the threshold for dividing the patients into groups A and B was set to 62.2%. Better repair (group A) was associated with more rapid proliferation and a healthier ultrastructure. The cells in group A showed stronger specific staining signifying osteogenic and chondrogenic differentiation; alkaline phosphatase (ALP) activity, an indicator of osteogenic differentiation, was higher in group A than in group B (OD, 2.39 ± 0.44 and 1.85 ± 0.52; *p* <  0.05).

**Conclusions:**

The quality of implanted stem cells is closely related to treatment efficacy and determines whether the defective self-repair in the necrotic area can be corrected to enhance repair and thus achieve the desired therapeutic outcome.

**Trial registration:**

The trial registration number: ChiCTR-ORC-17011698 (retrospectively registered at 2017-06-19).

## Background

Osteonecrosis of the femoral head (ONFH) is a common disease that greatly affects the quality of life of patients [1]. The course of this disease is progressive, and the arthritis severity gradually increases [2] until patients with late-stage ONFH require total hip arthroplasty (THA) [3]. In clinical practice, it is challenging to determine how to prevent the progression of early-stage ONFH or to completely repair the necrotic areas to avoid THA. Core decompression is one of the available early treatments [4] and can significantly relieve pain in patients with early-stage ONFH [5]. However, a considerable number of patients do not achieve a satisfactory outcome in terms of repair of the femoral head necrotic area [5]. Femoral head necrosis is a disease involving the activity of local stem cells in the femoral head [6]. In ONFH patients, the number and viability of mesenchymal stem cells (MSCs) in the femoral head were found to be decreased [7]. Therefore, stem cell implantation is a potential treatment strategy. MSCs are capable of self-renewal and differentiation into multiple lineages, including bone, cartilage, adipose tissue, muscle, and tendon [8]. Theoretically, implanted stem cells with self-renewal and multidirectional differentiation abilities could drive the repair of necrotic areas, compensating for the functional defects in local stem cells [9]. Both clinicians and patients have great hope for this approach. However, in practice, stem cell therapy combined with core decompression has only a slight advantage over core decompression alone [10], and it still does not achieve the anticipated therapeutic effects, as some postoperative patients show no apparent repair in the necrotic area [11]. Unfortunately, these patients who experience treatment failure have not only paid the expensive hospitalization fee but also endured psychological and physical pain. Hence, it is of great importance to determine the cause of this poor outcome. Differences in stem cell proliferation and differentiation capacities may be important factors affecting the efficacy of the combined therapy. Therefore, this study aimed to investigate the mechanism by which the quality and number of stem cells affect the correlation between stem cell viability and the repair efficacy of ONFH.

## Methods

### General data

The study was reviewed and approved by the University Ethics Committee. Written informed consent was obtained from all subjects.

This study included 19 men and 11 women with idiopathic ONFH (Arco stage II) and a mean age of 30.6 years. All patients received the following evaluations before surgery: Harris hip score (HHS), visual analogue scale (VAS) of pain, routine blood tests, liver and kidney function tests, comprehensive coagulation tests, erythrocyte sedimentation rate (ESR), C-reactive protein level, electrocardiogram (ECG), chest anteroposterior radiography, bilateral hip anteroposterior radiography, and bilateral hip plain magnetic resonance imaging (MRI).

### Harvest and isolation of autologous stem cells

Prior to surgery, patients received recombinant granulocyte colony-stimulating factor (GCSF, 30 IU IM qd × 5 days) to induce stem cell mobilization in the bone marrow [12]. The patient was placed in the supine position under anesthesia. Bone marrow aspiration from the iliac crest was performed, and 100 ml of bone marrow and 100 ml of peripheral blood were harvested. The bone marrow and blood were placed in heparin-coated centrifuge tubes and centrifuged twice at 4000 r/min for 10 min. A 30-ml cell suspension was obtained, and 15 ml of this suspension was added to a collagen sponge to generate a gel-like cell-material composite for surgery. The remaining 15 ml was used to assess cell number and viability.

### Mononuclear cell (MNC) count

MNCs were isolated by density gradient centrifugation at 3000 rpm for 30 min with a Percoll cell separator. After centrifugation, the solution was divided into three layers; the middle layer contained the MNCs, which were isolated and counted with a microscope eyepiece reticle.

### Isolation and culture of human bone marrow-derived MSCs (hBMSCs)

The cells were resuspended at a 1:1 ratio in the culture medium (Dulbecco’s modified Eagle medium (DMEM) supplemented with 10% fetal bovine serum (FBS), 100 units/mL penicillin, and 100 mg/mL streptomycin), placed in cell culture flasks at a density of 3 × 10^6^ cells/mL, and cultured in an incubator at 37 °C and 5% CO_2_. The cells were passaged at 80% confluence using a 0.2% trypsin solution. Cells at passage three (P3) were used for the subsequent experiments.

### Immunophenotypic characterization of hBMSCs

Cells at P3 were collected after digestion. A 100-μl suspension containing 1 × 10^6^ cells was immunostained for cell surface markers and analyzed using an Aria SE flow cytometer and Cell Quest Pro software. hBMSCs were identified as cells positive for CD105, CD73, CD44, and CD90 and negative for CD34, CD45, and HLA-DR. The cells were incubated with CD44-APC, CD90-FITC, CD105-CY5.5, CD73-PE, CD34-PE, CD45-PE, and HLA-DR-PE at the suppliers’ recommended dilutions for 45 min at room temperature in the dark. Flow cytometry was performed after two washes with phosphate-buffered saline (PBS).

### Ultrastructural observation

The cell ultrastructure was observed using transmission electron microscopy (TEM, Tecnai 10, FEI, Hillsboro, OR, USA). Specimen preparation for TEM was as follows: P3 cells were concentrated by low-speed centrifugation (2000 rpm). The cell pellets were prefixed in 2.5% glutaraldehyde and then rinsed three times with PBS. Subsequently, the cell pellets were postfixed with 1% osmium tetroxide, rinsed three times with PBS, and dehydrated in a series of acetone in distilled water (30, 50, 70, 90, 95, and 100%). Finally, the dehydrated cell pellets were embedded and sectioned with a diamond knife. The ultrathin specimen sections were stained with uranyl acetate and lead citrate for 30 min each and then observed by TEM.

### Quantitation of multilineage differentiation

Cells at P3 were trypsinized and plated onto 100 mm^2^ tissue culture plates at 10^5^ cells per plate. After the cells were incubated in the culture medium for 1 day, the medium was replaced with either osteogenic medium containing DMEM, 10 mM β-glycerophosphate, 0.1 M dexamethasone, 50 g/ml L-ascorbic acid 2-phosphate, and 10 g/ml insulin or chondrogenic medium containing DMEM, 1% (v/v) FBS, 10 ng/ml rh-TGFβ1, 50 mg/L ascorbic acid, 6.25 mg/ml insulin, 10^− 7^ M dexamethasone, 100 U/ml penicillin, 100 mg/ml streptomycin, and 2 mM L-glutamine. Culture media were replaced every 3 days. Cells were assessed at 14 days after inducing differentiation. The quantitative analysis of osteogenic differentiation was performed by measuring alkaline phosphatase (ALP) activity with an Alkaline Phosphatase Assay Kit (Abcam, Cambridge, MA) per the manufacturer’s protocol and by quantifying alizarin red S staining of calcified tissues according to a standard protocol. Toluidine blue (D8857, NobleRyder, China) staining was performed to evaluate chondrogenic differentiation.

### Image analysis

hBMSCs were stained with alizarin red S after 14 days of osteogenic induction and with toluidine blue after 14 days of chondrogenic induction. The area percentage of staining was determined individually by ImageJ version 1.50d. Images were first converted to a grayscale stack by selecting the RGB values, activating thresholding, and adjusting the region of interest based on the original colored image. This highlights the region of interest within the grayscale in red, and the area percentages were measured.

### Core decompression and stem cell composite implantation

**Fig. 1:**
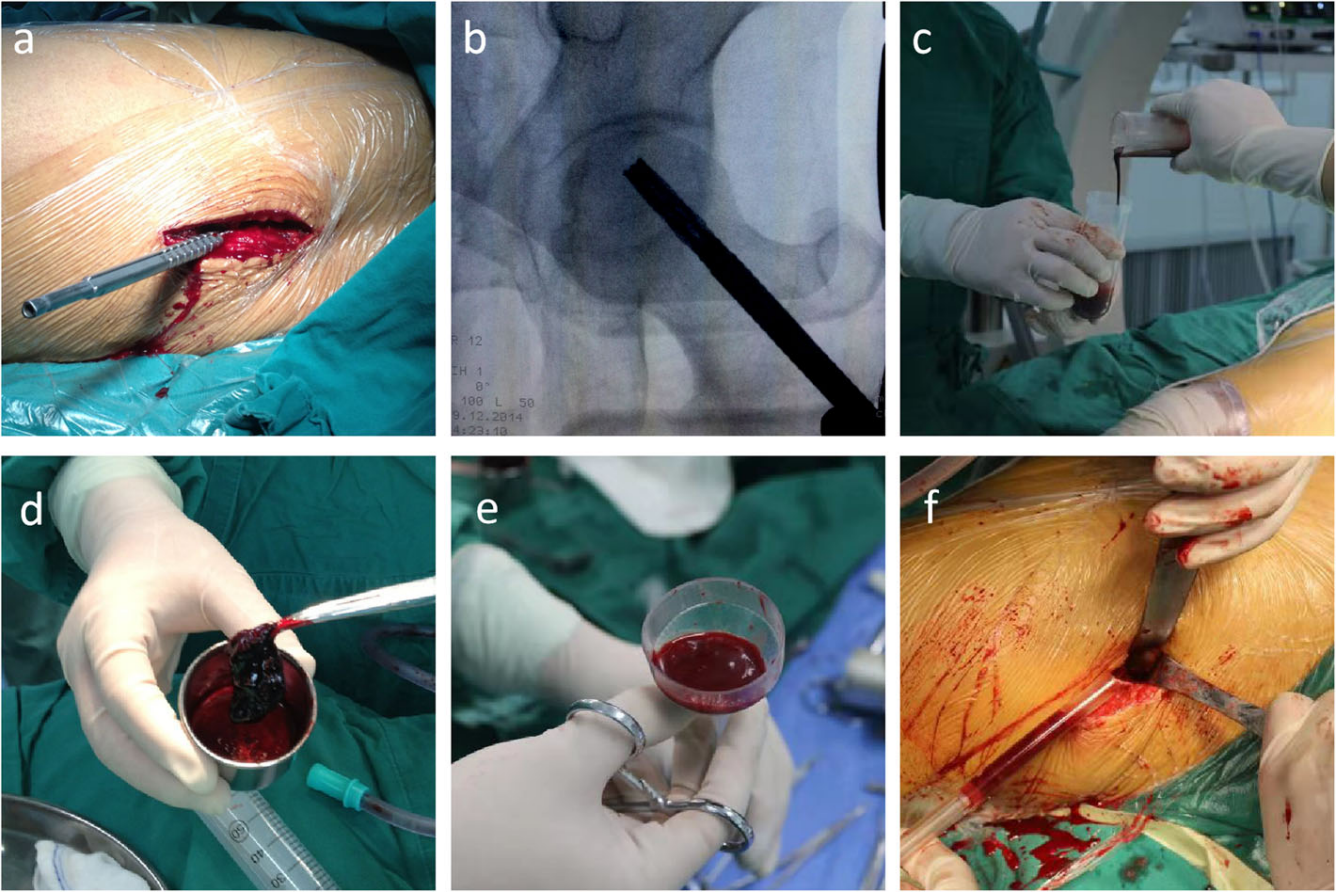
**a** Core decompression. **b** Intraoperative C-arm fluoroscopy. **c** Stem cell suspension obtained by centrifugation. **d** Collagen sponge-cell composites. **e** Implantation device for collagen sponge-cell composites. **f** Material implantation

Upon achieving successful anesthesia, a 3 cm incision was made below the greater trochanter. Under C-arm fluoroscopy guidance, a 2.5 mm Kirschner wire (K-wire) was drilled into the lesion site of the femoral head (2–3 mm beyond the subchondral level) via the femoral neck. A 6.5 mm drill was drilled into the same site over the K-wire. A customized long-handled curette was used to completely remove the lesion tissue beneath the cartilage. Fluoroscopy was performed to ensure complete lesion removal. Next, the collagen sponge-cell composites were injected to fill this site. A piece of muscle membrane was used to cover the filled site to prevent cell leakage. All procedures were performed by the same surgical team (Fig. 1a-f).

### Postoperative management and follow-up

The patients were instructed to walk with a supportive device for 9 months after surgery. The HHS and imaging studies of the treated hip were used to assess the clinical efficacy during follow-up. Patients’ pain assessments were scored on a VAS from 0 cm (no pain) to 100 cm (severe pain).

### Assessment criteria

HHS of the hip: The delta HHS was calculated as the difference between the HHS before surgery and the HHS at 9 months after surgery. A greater difference was believed to represent more significant functional improvement.

**Fig. 2:**
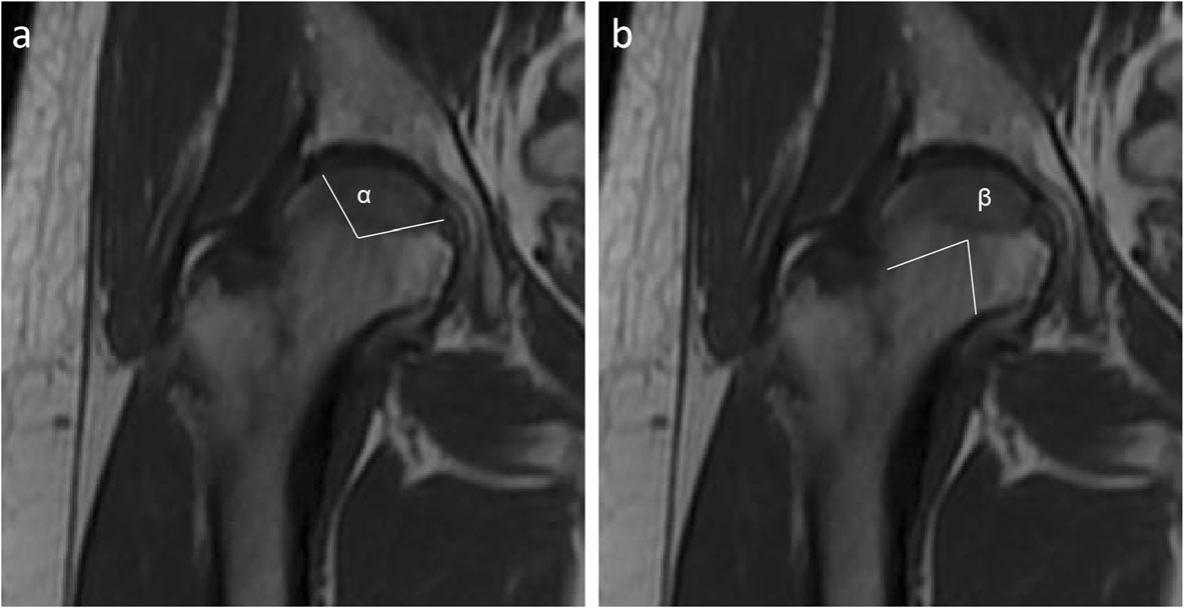
**a** The angle α was defined as the angle corresponding to the necrotic site on an MRI image. **b** The angle β was defined as the central angle corresponding to the femoral head in the same image (the angle between the connecting lines from the center of the femoral head to the femoral head-neck junction)

Necrotic area evaluation by MRI: A GE Signa 1.5 T superconducting MR (USA) was used for the hip examination. The coronal T1-weighted images were selected to measure the necrotic area angle α and the central angle β of the femoral head (Fig. 2a, b). The necrotic area ratio (before vs after treatment) of each hip was determined according to FengChao Zhao’s method [13]. The repair ratio was calculated according to the following formula, and a higher repair ratio indicates more significant lesion repair.

$$ \mathrm{Repair}\ \mathrm{ratio}\ \left(\%\right)=\frac{\mathrm{necrotic}\ \mathrm{area}\ \mathrm{ratio}\ \mathrm{before}\ \mathrm{surgery}-\mathrm{necrotic}\ \mathrm{area}\ \mathrm{ratio}\ 9\ \mathrm{months}\ \mathrm{after}\ \mathrm{surgery}}{\mathrm{necrotic}\ \mathrm{area}\ \mathrm{ratio}\ \mathrm{before}\ \mathrm{surgery}}\times 100\% $$

The mean repair ratio was set as the threshold to divide the patients into group A (ratio above the mean) and group B (ratio below the mean). The ultrastructure, proliferative capacity, and multidirectional differentiation ability were compared between the groups.

### Statistical analysis

SPSS version 21.0 was used for statistical analyses. The paired t test, nonpaired t test, and Spearman correlation analysis were used. All tests were two-tailed at the 5% significance level.

## Results

### Necrotic area ratio as determined by MRI

**Fig. 3:**
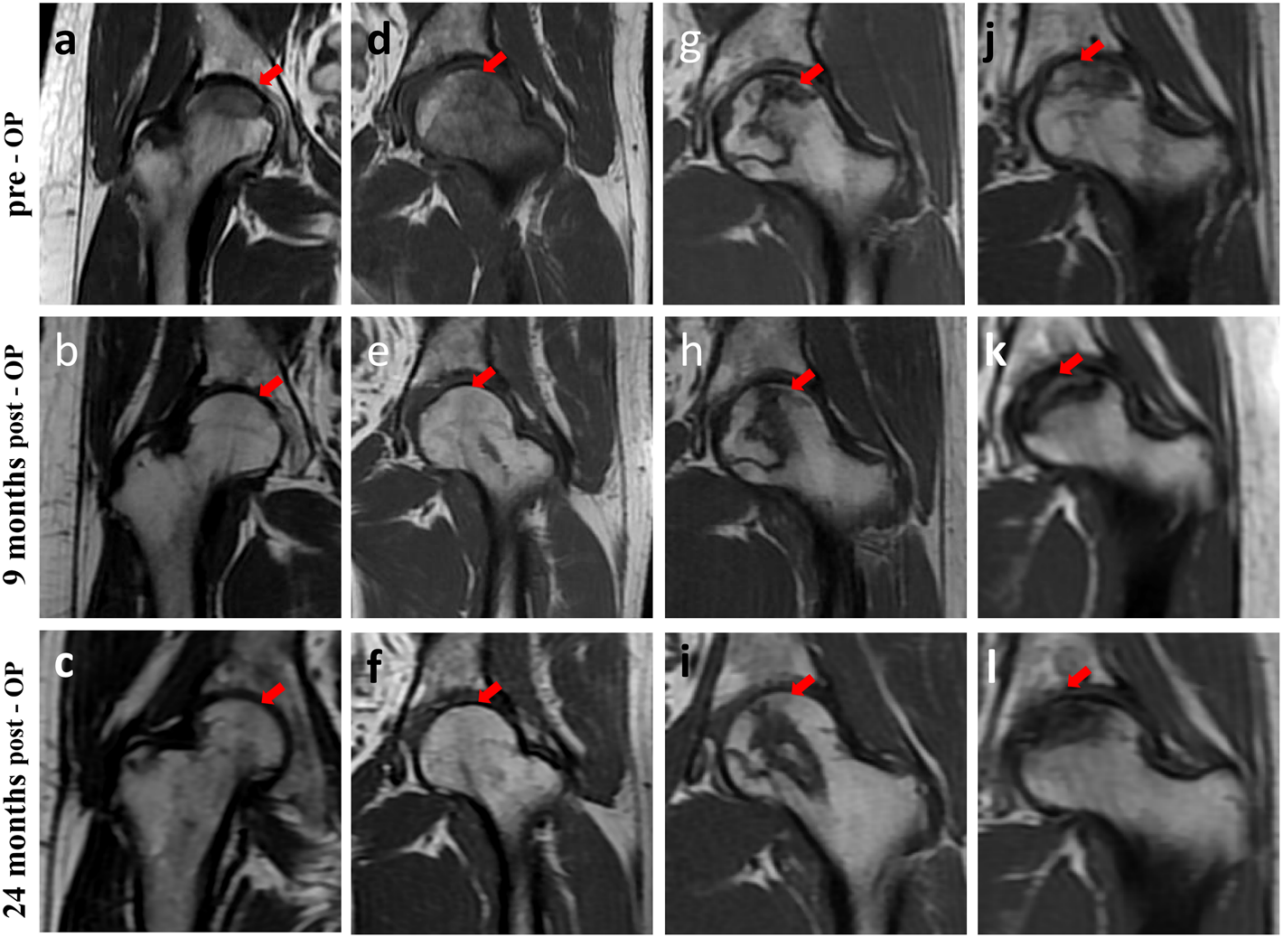
**a-l** T1-weighted MRI images of the necrotic area before and after the operation. Arrow Location of the necrotic area. **a-f** In some patients, the necrotic area ratio was significantly lower at 9 months after surgery than before surgery. **g-l** In other patients, the necrotic area ratio was not significantly lower at 9 months after surgery than before surgery. In all patients, the necrotic area did not change considerably from 9 months to 24 months after the operation

**Fig. 4:**
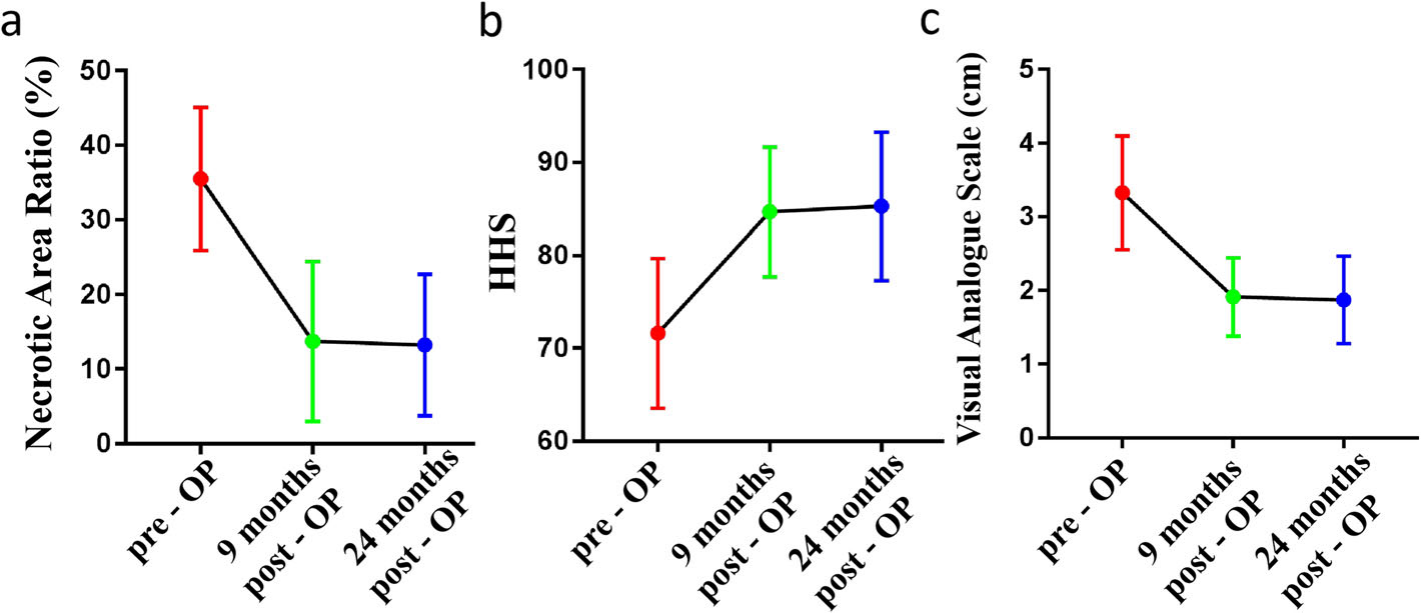
**a** The necrotic area ratio (%) was significantly lower at 9 months after surgery than before surgery (*p* < 0.05). The necrotic area was not significantly different at 9 months and 24 months after the operation. **b** The HHS was significantly higher at 9 months after surgery than before surgery (*p* < 0.05). **c** The VAS score was significantly lower at 9 months after the operation than before the operation (*p* < 0.05). The VAS was slightly lower at 24 months after the operation (1.87 ± 0.60) than at 9 months after the operation (*p* > 0.05)

This ratio significantly decreased from (35.51 ± 9.57)% before surgery to (13.74 ± 10.70)% at 9 months after surgery. At 24 months after the operation, the necrotic area ratio was (13.24 ± 9.49)%, which was not significantly different from that at 9 months after the operation (*p* >  0.05) (Figs. 3a-l and 4a).

### HHS and VAS of the hip

The HHS significantly increased from (71.63 ± 8.05) before surgery to (84.66 ± 6.97) at 9 months after surgery (*p* <  0.05). The HHS was slightly higher at 24 months after the operation (85.27 ± 7.97) than at 9 months after the operation (*p* >  0.05). The delta HHS (13.04 ± 5.86) was obtained by subtracting the score at 9 months after surgery from the preoperative score (Fig. 4b). The VAS significantly decreased from (3.33 ± 0.77) before surgery to (1.91 ± 0.53) at 9 months after surgery (*p* < 0.05). Moreover, the VAS was slightly lower at 24 months after the operation (1.87 ± 0.60) than at 9 months after the operation (*p* >  0.05) (Fig. 4c).

### Correlation between the repair ratio and the delta HHS

An increased repair ratio was associated with a greater HHS (Table 1), suggesting that the extent of lesion repair is correlated with the extent of functional improvement. Based on the mean repair ratio of (62.2 ± 27.0)%, the threshold for dividing the patients into groups A and B was set at 62.2% (Table 2). There were no significant differences in baseline characteristics between the two groups (Table 3).

**Table 1:** Correlations among repair ratio, delta HHS, and age

Parameter	Repair ratio	
	r value	*p* value
Delta HHS	0.850	< 0.05
Age (years)	0.084	> 0.05

*p* < 0.05 was considered to indicate statistical significance

**Table 2:** Repair ratio (%) in groups A and B

N	Group A	Group B
1	62.5	8.7
2	62.8	12.4
3	67.2	18.7
4	69.1	23.7
5	75.3	35.9
6	76.4	38.1
7	84.2	38.5
8	85.7	40.7
9	87.3	42.3
10	87.8	49.7
11	88.5	53.1
12	91.9	55.7
13	94.2	57.3
14	96.5	60.4
15	100	
16	100

**Table 3:** Baseline patient characteristics

Variable	Group A (*N* = 16)	Group B (*N* = 14)	*p* value
Side of treated hip			
Left (%)	10 (62.5%)	6 (42.9%)	> 0.05
Right (%)	6 (37.5%)	8 (57.1%)	> 0.05
Age (mean ± SD)	30.69 ± 5.87	30.43 ± 4.45	> 0.05
Gender (male/female)	10/6	9/5	> 0.05
Preoperative HHS (mean ± SD)	68.67 ± 8.38	75.01 ± 6.37	> 0.05
Preoperative necrotic area ratio (%)	34.17 ± 9.01	37.05 ± 10.29	> 0.05
MNC concentration (× 10^9^/L, mean ± SD)	9.94 ± 1.46	10.04 ± 1.47	> 0.05
Hospitalization expense ($)	3203.31 ± 115.23	3190.14 ± 134.37	> 0.05

No variables were significantly different between groups A and B at baseline

### Ultrastructural characteristics of hBMSCs and the duration of cell growth before passage

**Fig. 5:**
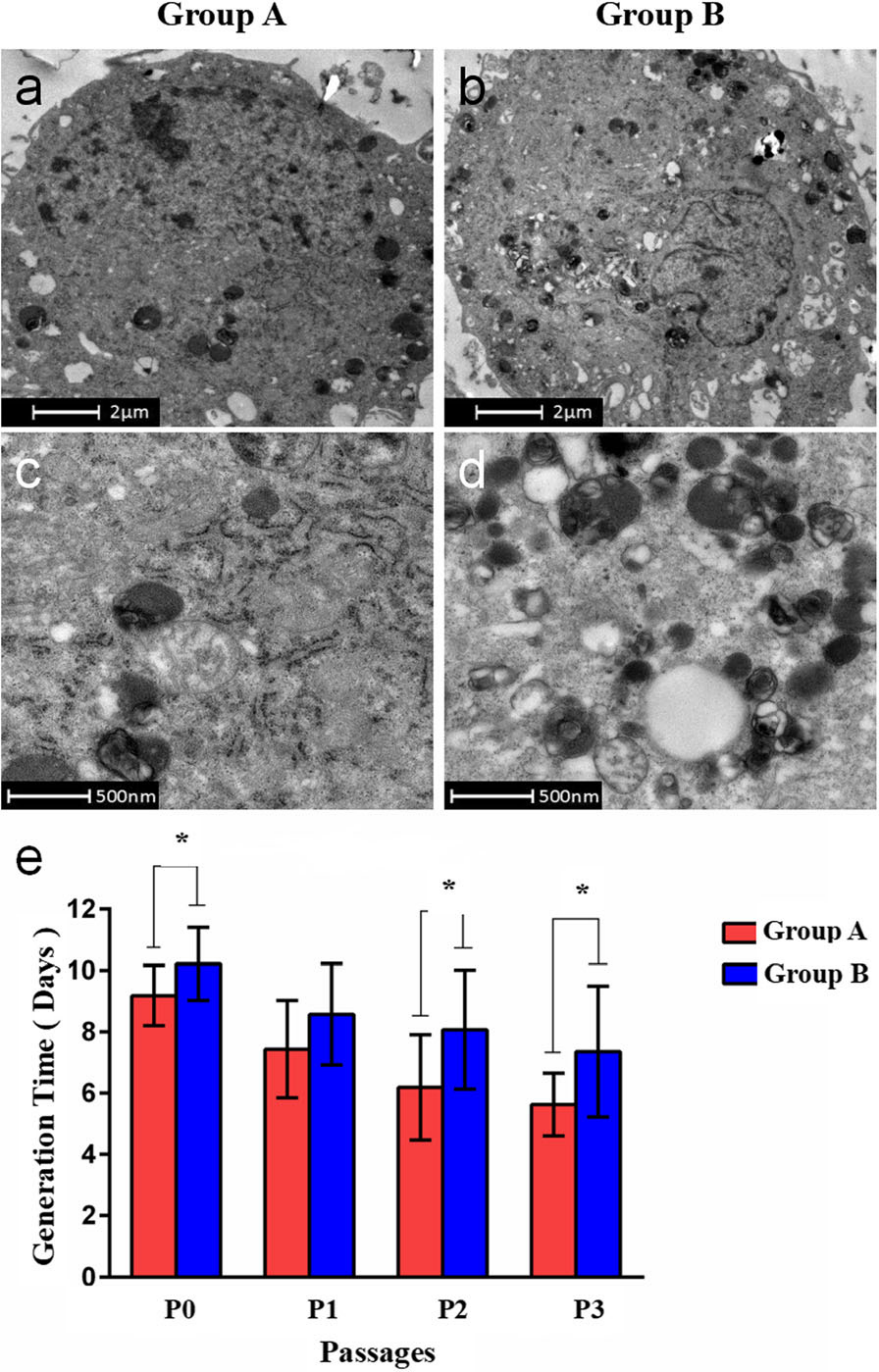
**a-d** The hBMSCs from group A had large nuclei and large, obvious nucleoli with an even heterochromatic distribution and rich cytoplasm with intermediate electron density. The hBMSCs from group B had decreased cytoplasmic electron density and numerous vacuoles and autophagosomes of varying size. **e** Comparison of the time between passages of hBMSCs in groups A and B. * *p* < 0.05

The hBMSCs from group A exhibited large, irregular, round or oval nuclei with an intact nuclear membrane and large, obvious nucleoli with an even heterochromatic distribution. The cells were rich in cytoplasm with an intermediate electron density. The organelles, such as the rough endoplasmic reticulum, Golgi apparatus, and mitochondria, were normal and abundant with a clear structure. The hBMSCs from group B had decreased electron density in the cytoplasm and plentiful vacuoles and autophagosomes of varying size. The autophagosomes contained incompletely digested residual organelles, cytoplasmic components, and ruptured mitochondria (Fig. 5a-d). The cell ultrastructure analysis showed more characteristics of healthy cells in group A compared with group B. The duration of cells in P0 was 9.19 ± 0.98 days in group A and 10.21 ± 1.19 days in group B (*p* < 0.05). The duration in P2 decreased to 6.19 ± 1.72 days in group A and 8.07 ± 1.94 days in group B (*p* < 0.05), and that in P3 was 5.63 ± 1.03 days in group A and 7.36 ± 3.13 days in group B (*p* < 0.05). The times spent in P0, P2, and P3 were significantly shorter in group A than in group B (*p* < 0.05), but there was no significant difference in P1 duration between groups A and B (Fig. 5e).

### Cell surface marker expression

**Fig. 6:**
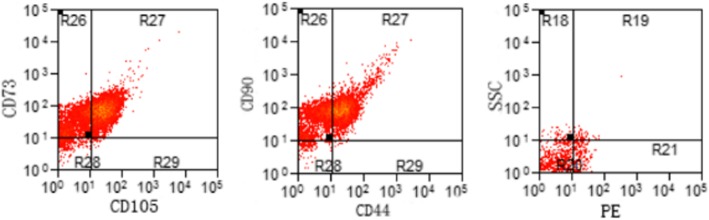
Flow cytometry results. The cells highly expressed CD105, CD73, CD44, and CD90 but not CD34, CD45, or HLA-DR.

Flow cytometry was used to detect surface antigen expression on P3 hBMSCs in groups A and B. The analyzed cells were highly positive for CD105, CD73, CD44, and CD90 but were negative for the hematopoietic stem cell markers CD34, CD45, and HLA-DR (Fig. 6).

### Multilineage differentiation

**Fig. 7:**
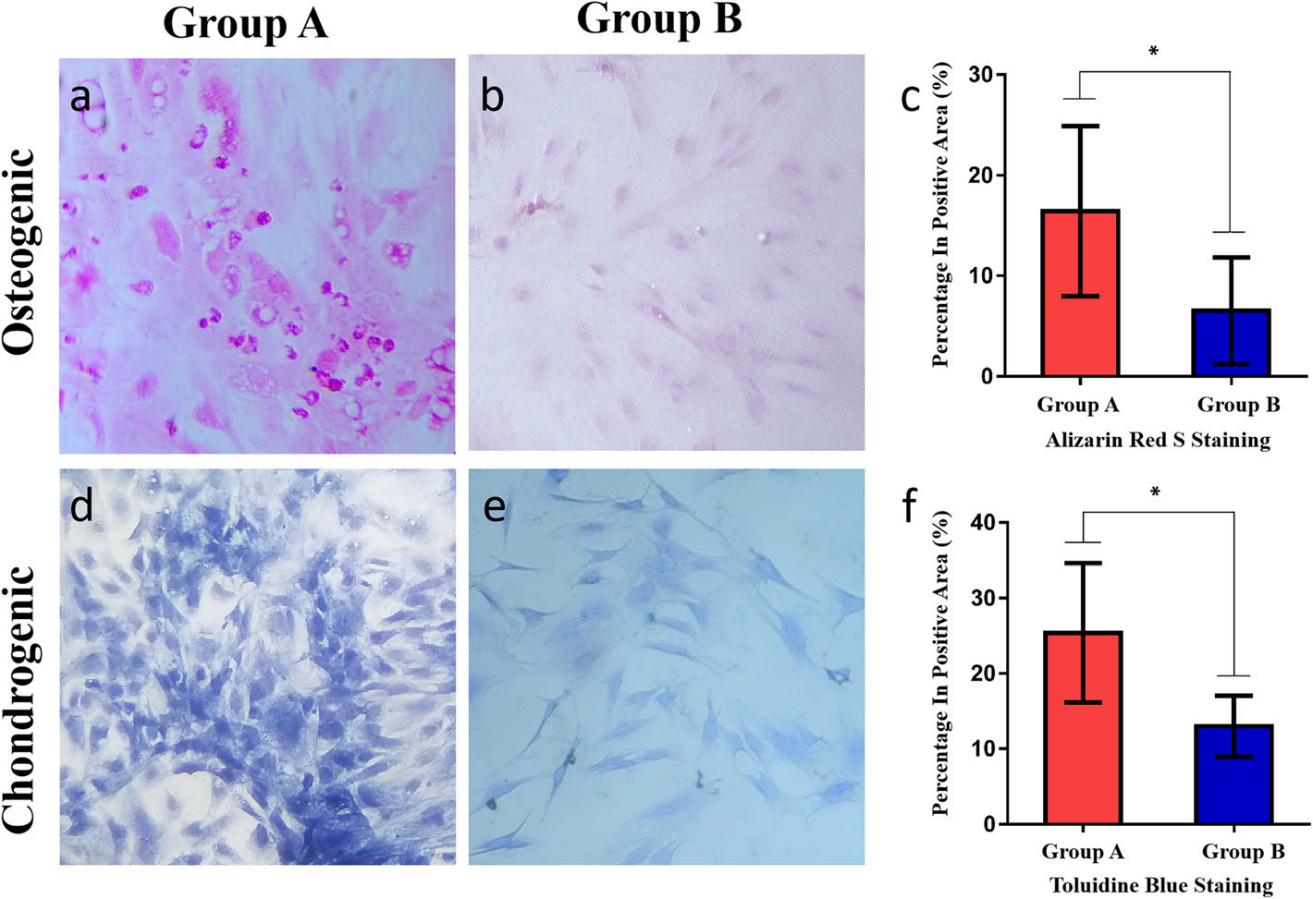
**a-f** Comparison of multilineage differentiation. **a**, **b** Alizarin red S staining (× 100) after a 14-day osteogenic induction of hBMSCs. **d**, **e** Toluidine blue staining (× 100) after a 14-day chondrogenic induction of hBMSCs. **c**, **f** The mean positive area percentage was significantly higher in group A than in group B

**Fig. 8:**
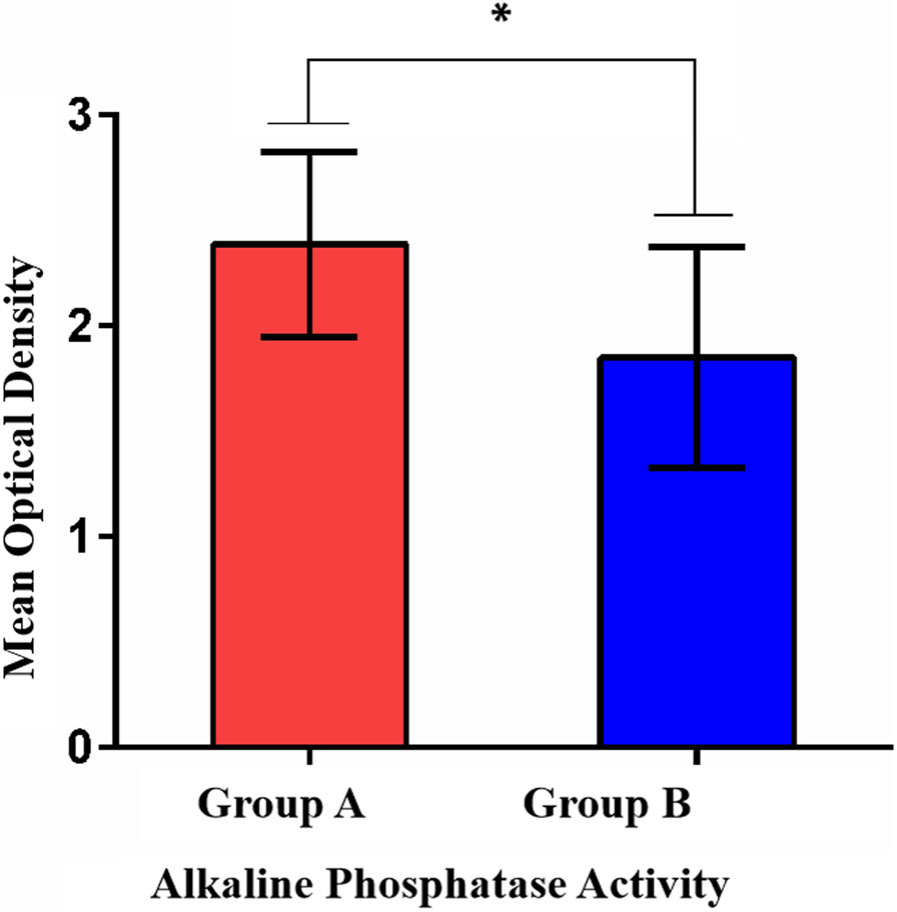
ALP activity after the induced differentiation of hBMSCs from groups A and B. * *p* < 0.05

After a 14-day induction, hBMSCs in the two groups showed different degrees of osteogenic and chondrogenic differentiation. Cells in group A were more strongly stained than those in group B (Fig. 7a-f). The hBMSCs in group A had higher ALP activity after osteogenesis induction than those in group B (OD, 2.39 ± 0.44 vs 1.85 ± 0.52; *p* < 0.05) (Fig. 8).

Alizarin red S staining appears as red calcium nodule staining, whereas toluidine blue staining appears as blue granular cytoplasmic staining. Using ImageJ, the positive staining area percentages were calculated to be 16.44 ± 8.48 in group A and 6.52 ± 5.31 in group B for alizarin red S (Fig. 7c) and 25.39 ± 9.24 in group A and 12.99 ± 4.08 in group B for toluidine blue (Fig. 7f). Group A had a significantly higher mean positive area percentage than group B for both stains (*p* < 0.05).

## Discussion

Based on the results of this study, we can conclude that the efficacy of autologous stem cells combined with core decompression for the treatment of early-stage ONFH is associated with autologous stem cell viability. The use of hBMSCs with greater proliferation and differentiation capacities can improve treatment efficacy.

The traditional treatment for early-stage ONFH is core decompression [14], which can release pressure and open the small vessels blocked by pressure in the femoral head, thus relieving pain [15]. However, imaging data from postoperative follow-ups showed that the necrotic area of the femoral head did not shrink significantly in a number of patients [16] and even continued to expand in some cases [17], which eventually led to the collapse and deformation of the femoral head [5]. Therefore, core decompression alone cannot achieve reconstruction and satisfactory repair of the femoral head necrosis area [18]. However, stem cells offer hope. Sugaya’s team confirmed the efficacy of local BMSC transplantation in the treatment of femoral head necrosis through animal experiments; they showed that BMSCs in the necrotic area can survive, proliferate, differentiate into bone, and promote repair [19, 20]. However, stem cell therapy for osteonecrosis has not shown sufficient repair outcomes in clinical application [7]. Wojciech Pepke compared the individual efficacy of bone marrow cell implantation and core decompression in the treatment of early-stage ONFH and found no significant difference between treatments in the postoperative change in necrotic area [11]. The activity and quantity of stem cells are key factors that influence the therapeutic effect.

The HHS was significantly lower 9 months after the operation than before the operation. From 9 months to 2 years post operation, hip function continued to improve in some patients but deteriorated in others; nonetheless, the overall trend was stable. Valérie Gangji followed up 13 patients and reported similar results [21]. The VAS score 9 months after surgery was significantly lower, and the trend was similar to that of the functional score. There was no significant change in the VAS score at 2 years after the operation. Tabatabaee RM also obtained similar results [22]. In this study, diversity in the regional repair of femoral head necrosis was observed. The HHS changed significantly in patients with better repair, which indicated that the repair of the necrotic area was closely related to improved function [12]. There was no significant change in the necrotic area from 9 months to 2 years after the operation, indicating that the repair and reconstruction of the necrotic area mainly occurred within 9 months, which is similar to the fracture healing period [23]. Therefore, the extent of repair of the necrotic area at 9 months after the operation could indicate the therapeutic effect of stem cell implantation.

In 1999, Hernigou reported a decrease in the activity of stem cells in ONFH [24]. This decreased activity may affect local tissue and vascular regeneration, oxygen supply, and osteogenic function, eventually leading to ONFH [25]. Moreover, decreased stem cell activity can hinder the repair of necrotic areas, thus forming a vicious cycle [26]. Therefore, it is necessary to improve the growth and differentiation capacities of stem cells in the necrotic area of the femoral head [27, 28]. Implanted hBMSCs can differentiate into various types of cells with vascularization and osteogenesis functions to promote the repair of the necrotic areas [29]. The concept of combining stem cell transplantation with core decompression for the treatment of early-stage ONFH was first proposed by Hernigou in 2002 [30]. He observed that the number of transplanted stem cells was closely related to prognosis. In a 2005 report on the evaluation of stem cell transplantation for the treatment of bone nonunion and osteonecrosis, the authors proposed that the key to effective stem cell transplantation is a stem cell concentration greater than 2 million MNCs per ml [31]. However, the differentiation capacity of the implanted stem cells was not discussed in this previous study or others.

This study aimed to discuss the relationship between the efficacy of core decompression combined with stem cell transplantation and the proliferation and differentiation capacities of the implanted stem cells in patients with idiopathic ONFH. Using TEM, we found that implanted hBMSCs with poor repair capacity had decreased cytoplasmic electron density and numerous vacuoles and autophagosomes of varying size; these autophagosomes contained incompletely digested residual organelles and cytoplasmic components. Based on the morphology of the vacuoles, we speculated that they may be derived from mitochondrial vacuolization, which can affect aerobic respiration [32] and further compromise the capacity for cell proliferation and differentiation [33]. The presence of autophagosomes and digested myelin-like bodies in numerous cells indicate cell aging and the initiation of self-protective responses [34, 35]. We speculate that abnormal changes in organelles (e.g., mitochondria) may affect the synthesis of certain enzymes important for cell behavior and metabolism [36], which is consistent with our finding that these cells took longer to reach the confluence necessary for passaging, indicating that metabolic abnormalities affect the proliferative activity of cells.

The osteogenic- and chondrogenic-specific induction assays also showed better outcomes in group A. In particular, after the osteogenic induction of hBMSCs, the ALP activity assay and alizarin red S staining showed lower osteogenic activity in cells from patients with poor necrotic area repair than in those from patients with good repair. Because it is essential for hBMSCs to promote healing [28], the osteosynthesis defects partially explain the poor repair of necrotic areas following stem cell implantation. This defect may be a causative agent of femoral head necrosis in these patients [37].

In this study, the viability of hBMSCs from patients was observed in the two groups generated based on the repair ratio. The reason for this grouping is that greater necrotic area repair indicates a better treatment effect. There is no clear value that defines the curative effect and no related research indicating the extent of necrotic area repair that produces satisfactory clinical results.

The total cost of stem cell therapy combined with core decompression is approximately 3200 dollars, while the cost of determining stem cell viability is approximately 170 dollars. Evaluating cell viability before the operation has great predictive value for this combined therapy, provides a theoretical basis for treatment decisions and avoids additional economic burdens and psychological and physical pain caused by ineffective therapy.

In summary, core decompression combined with stem cell transplantation is currently a popular treatment option for early-stage ONFH. However, the use of undifferentiated stem cell therapy and the lack of quality assessment of the implanted cells are likely to compromise the expected treatment efficacy, which can increase the economic burden on patients and even delay disease diagnosis and treatment. In future clinical work, an initial examination of the quality and quantity of peripheral blood and bone marrow cells needs to be performed. In cases with a limited number of stem cells or poor stem cell osteogenesis activity, the regimen should be adjusted. A next priority is to identify a more effective, noninvasive, simple, and inexpensive preoperative stem cell assessment method.

Due to the small sample size and short follow-up period in this study, the best indications and long-term efficacy of this treatment need to be determined in additional studies.

## Conclusion

The efficacy of core decompression combined with autologous stem cell transplantation for the treatment of early-stage ONFH is closely related to stem cell viability.

## Data Availability

The datasets generated and analyzed during the current study are not publicly available but are available as deidentified data sheets from the corresponding author on reasonable request.

## Abbreviations

ALP: Alkaline phosphatase
BMSCs: Bone marrow-derived mesenchymal stem cells
DMEM: Dulbecco’s modified Eagle medium
FBS: Fetal bovine serum
GCSF: Granulocyte colony-stimulating factor
HHS: Harris hip score
MNCs: Mononuclear cells
MRI: Magnetic resonance imaging
OD: Optical density
ONFH: Osteonecrosis of the femoral head
